# Genetics and Function of Neocortical GABAergic Interneurons in Neurodevelopmental Disorders

**DOI:** 10.1155/2011/649325

**Published:** 2011-08-18

**Authors:** E. Rossignol

**Affiliations:** ^1^Department of Pediatrics, Neurology, Sainte-Justine Hospital and Research Center, 3175 Chemin de la Côte Sainte-Catherine, Montreal, QC, Canada H3T 1C5; ^2^Department of Pediatrics, Brain Disease Research Group, Sainte-Justine Hospital and Research Center, 3175 Chemin de la Côte Sainte-Catherine, Montreal, QC, Canada H3T 1C5

## Abstract

A dysfunction of cortical and limbic GABAergic circuits has been postulated to contribute to multiple neurodevelopmental disorders in humans, including schizophrenia, autism, and epilepsy. In the current paper, I summarize the characteristics that underlie the great diversity of cortical GABAergic interneurons and explore how the multiple roles of these cells in developing and mature circuits might contribute to the aforementioned disorders. Furthermore, I review the tightly controlled genetic cascades that determine the fate of cortical interneurons and summarize how the dysfunction of genes important for the generation, specification, maturation, and function of cortical interneurons might contribute to these disorders.

## 1. Introduction

The exquisite complexity of cognitive functions stems from tightly regulated interactions between distributed cortical networks performing precise neural computations. GABAergic inhibitory interneurons (INs), which represent a minority of neocortical neurons (20% in rodents [1]), play a crucial role in these cortical circuits. GABAergic INs shape the responses of pyramidal cells to incoming inputs, prevent runaway excitation, refine cortical receptive fields, and are involved in the timing and synchronisation of population rhythms expressed as cortical oscillations [2–9]. Consequently, disruption of cortical GABAergic IN function has been linked to various neurodevelopmental disorders, including epilepsy, mental retardation, autism, and schizophrenia [10–15]. 

**Figure 1:**
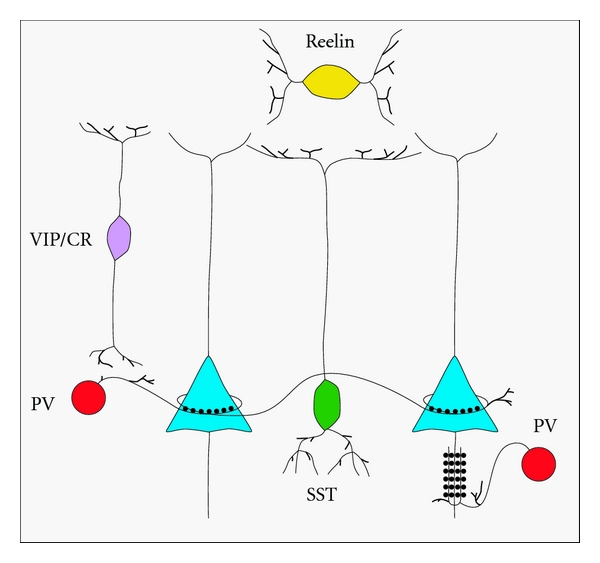
Interneuron diversity. Interneurons are diverse in terms of their histochemical profile, morphology, physiological properties, and connectivity. In this schematic representation, parvalbumin-positive (PV) interneurons (red) include basket cells forming perisomatic contacts on adjacent pyramidal cells (dark blue), as well as chandelier cells that target the pyramidal cell axon initial segment. Somatostatin-positive (SST) interneurons include Martinotti cells that contact pyramidal cell dendrites in layer I. Vasointestinal peptide (VIP) and calretinin (CR) double-positive bitufted interneurons target pyramidal cells and other interneurons. Neurogliaform cells, marked with reelin, are the most abundant interneurons in layer I and provide tonic GABAergic inhibition via volume transmission of GABA.

Cortical INs are diverse in terms of their anatomical laminar distribution, histochemical marker expression, intrinsic physiological properties, and connectivity (Figure 1) [5, 6, 9, 16–22]. This heterogeneity is characterized by the expression of specific combinations of ion channels, receptors, and membrane cell adhesion molecules [7]. These specific protein expression profiles are the result of tightly controlled genetic pathways that regulate cortical IN identity [8, 23–29]. Anomalies in these genetic pathways might therefore underlie some of the neurodevelopmental and neurocognitive disorders seen in humans. In the current paper, I will give an overview of cortical IN diversity, summarise the various roles of cortical INs in neuronal circuit development and function, review the genetic pathways involved in specifying cortical GABAergic IN diversity, and explore the pathological correlates of genetic anomalies leading to interneuron dysfunction in rodents and humans. As the current paper focuses on neocortical INs, readers are directed to other sources for a broader description of other GABAergic populations, including those of the amygdala, striatum, hippocampus, thalamus, and olfactory bulbs, which also participate in the corticolimbic and corticosubcortical circuits involved in cognition and emotional processing [7, 30–40].

### 1.1. Diversity of Cortical GABAergic Interneurons Subtypes and Roles

Neocortical GABAergic INs are heterogeneous, and different subtypes of INs have different spatial and temporal origins. As a group, neocortical INs are derived from transient ventral telencephalic structures referred to as the ganglionic eminences [27, 29, 41–46] as well as from the preoptic area [47]. The medial ganglionic eminence (MGE) produces approximately 70% of neocortical INs, including the parvalbumin-positive (PV) fast-spiking interneurons and the somatostatin-positive (SST) interneurons, which represent 40% and 30% of all neocortical INs, respectively [27, 46, 48]. By contrast, the caudal ganglionic eminence (CGE) gives rise to the remaining 30% of neocortical INs, a more heterogeneous group of cortical INs that share the unique expression of 5HT3A ionotropic serotoninergic receptors, rendering them highly responsive to the neuromodulatory effects of serotonin [9, 43, 46, 48, 49]. A majority of CGE-derived interneurons belong to either the reelin-positive multipolar population (including the late-spiking neurogliaform cells), the vasointestinal-peptide- (VIP-) positive bitufted population (including a calretinin- (CR)-positive population), or the VIP-positive, calretinin-negative bipolar population. Finally, the preoptic area contributes a small portion of neocortical INs (<3%) that are not labelled by the usual interneuron markers mentioned above [47]. The lateral ganglionic eminence (LGE) mainly produces olfactory bulb and amygdalar INs, as well as striatal and nucleus accumbens medium spiny neurons, but is generally thought not to give rise to cortical INs [23, 33, 38, 41, 50, 51]. Different subtypes of cortical INs are identified based on their immunohistochemical, morphological, physiological, and connectivity properties, and they mediate different functions in mature networks as detailed below.

### 1.2. Parvalbumin-Positive Basket Cells

PV-positive interneurons include the perisomatically targeting basket cells and the less abundant axon-initial segment-targeting chandelier cells. PV-positive basket cells can be further divided according to various morphological characteristics, including somatic diameter, firing properties, and extent of dendritic and axonal arborisation [5, 9, 52, 53]. As a group, PV-positive basket cells display many characteristics which render them one of the fastest and most reliable sources of inhibition in the cortex. They exhibit low input resistance, fast membrane kinetics, brief action potentials with large afterhyperpolarisation, and minimal spike adaptation and are able to sustain high frequency firing rates [5, 18, 19, 21, 44, 52, 54]. These fast kinetics are partly due to their expression of Kv3 voltage-gated potassium channels [52, 55–59], which ensure quick repolarisation and termination of action potentials. In addition, PV-positive fast-spiking cells mediate fast reliable neurotransmission, as they rely mainly on P/Q-type presynaptic Ca^2+^ channels for tight coupling between action potentials and neurotransmitter release [60–63]. Furthermore, these PV-positive basket cells might be able to buffer calcium more efficiently, as they express high levels of Ca^2+^-binding proteins, including parvalbumin and calbindin. It is possible that this expression of Ca^2+^-binding proteins renders these cells more resistant to Ca^2+^-induced excitotoxicity in the face of high firing rates. 

PV-positive INs are the main inhibitory target of thalamocortical projections in the cortex. In addition, PV-positive basket cells form intricate nests of synaptic contacts on the soma of adjacent pyramidal cells, giving them rapid control over the excitability of their pyramidal cell targets. These INs are therefore well positioned to provide strong and fast feedforward inhibition to adjacent pyramidal cells, limiting the time window for temporal summation of excitatory inputs and spike generation within populations of pyramidal cells. This feature sharpens the cortical response and prevents runaway excitation following thalamocortical excitation [64–68].

In addition, PV-positive basket cells are highly interconnected with one another through both chemical and electric synapses (gap junctions), creating a vast web of synchronously active INs [69, 70]. This network of inhibitory INs triggers and maintains high-frequency gamma oscillations within ensembles of cortical pyramidal cells [69, 71–77]. In support of this, the loss of connexin32, which forms gap junction connections between PV-positive INs, results in the partial loss of task-induced gamma oscillations [78]. Gamma oscillations are important for the maintenance of attention, working memory, and the refinement of executive functions in humans and rodents [79–83]. Therefore, PV-positive interneuron dysfunction has been postulated to underlie the loss of gamma oscillations in schizophrenic patients displaying working memory and executive function abnormalities [14, 82, 84, 85].

### 1.3. Parvalbumin-Positive Chandelier Cells

Like PV-positive basket cells, PV-positive chandelier cells display brief nonadapting trains of action potentials upon stimulation and are able to sustain high frequency firing rates [17, 86, 87]. They are characterised morphologically by their cartridges of vertically oriented candlestick-like axonal arbors [88–90]) forming synapses on the axon initial segment (AIS) of pyramidal cells [89, 91]. Chandelier cells are unusual among interneurons in that their output has been postulated to be excitatory rather than inhibitory. Indeed, the stimulation of chandelier cells triggers depolarisations in target pyramidal cells in the cortex and dentate gyrus [92–95]. This has been attributed to the high concentration of chloride and elevated GABA_A_ reversal potential at the AIS, due to efficient Cl^−^ import by the NKCC1 transporter in the absence of the KCC2 transporter (see below) [92, 93]. However, it is still unclear whether such depolarizing responses are obligatorily excitatory [95]. Furthermore, in other circuits, such as in the CA1 region of the hippocampus, chandelier cells appear to trigger hyperpolarising responses [96]. Overall, the net effect of chandelier cells might be dependent on the local state of network activity and on the particular ion channel composition of local pyramidal cells in different brain regions. In vivo, chandelier cells might be involved in the generation of specific oscillatory activities as they fire immediately before hippocampal pyramidal cells during sharp-wave-associated ripples [97].

### 1.4. Somatostatin-Positive (SST) Interneurons

Somatostatin-positive interneurons, including Martinotti cells and non-Martinotti cells, are heterogeneous in terms of their immunohistochemical profile (variable colabelling with calretinin and calbindin), morphology (multipolar, bipolar, or unipolar), axonal projections (most target pyramidal cell dendrites in layer I but some project locally within their cortical layer), and intrinsic electrophysiological properties [9, 45, 98–100]. A majority of SST interneurons (including the Martinotti cells) share some physiological characteristics, including a low spike threshold, prominent after-hyperpolarisation, and spike rate adaptation. However, these cells differ in their spiking pattern at threshold (regular versus bursting), especially when fired from hyperpolarized step currents [17, 98, 99]. In general, compared to fast-spiking basket cells, SST-positive interneurons tend to be more excitable: they display a lower spike threshold and have a higher resting membrane potential [101]. One exception to this rule is a population of non-Martinotti cells, which have a high firing threshold, higher firing rate, shorter spike half width, and lower input resistance [45, 99]. These cells have been mostly described in layer 4 of the cortex and are preferentially labelled in the X94 GAD67-GFP transgenic line [9, 99]. 

SST-positive Martinotti cells are found across cortical layers II-VI but are most abundant in cortical layer V. They project vertically towards layer I where they contact pyramidal cell dendrites and extend multiple axonal collaterals towards adjacent cortical columns [19, 102, 103]. Martinotti cells regulate pyramidal cell excitability by controlling the dendritic summation and integration of synaptic inputs and sharpening the coding of stimulus intensity [104]. Furthermore, as their connectivity is simultaneously divergent, convergent, and recurrent, they mediate disynaptic inhibition between interconnected pyramidal cells as well as recurrent feedback inhibition onto presynaptic pyramidal cells [105, 106]. They are therefore well suited to prevent excessive and recurrent excitation within cortical networks. Furthermore, they are increasingly recruited by sustained stimulation, owing to the fact that the synapses that they receive from pyramidal cells are in most cases facilitating [101, 107]. This renders them good candidates to dampen excitation during high activation states. Dysfunction of somatostatin cells has therefore been postulated to underlie some forms of experimental or poststatus epilepticus seizure disorders [108].

Because of their expression of low-threshold voltage-gated calcium channels and persistent sodium currents, about 40% of SST cells display intrinsic bursting abilities and might act as pacemaker cells, thereby triggering particular cortical oscillations [224]. Indeed, SST-positive cells, which are highly interconnected via gap junctions, tend to oscillate spontaneously in the theta range (3–9 Hz) when stimulated electrically or with cholinergic agonists in vitro [101]. They could therefore be involved in pacing cortical pyramidal cells in the theta range.

### 1.5. Vasoactive Intestinal Peptide- (VIP-) Positive Interneurons

CGE-derived interneurons tend to populate more superficial cortical layers than MGE-derived interneurons. Approximately 40% of CGE INs express VIP, and these cells tend to be enriched in layers II/III [19, 46, 48, 225, 226]. VIP-positive INs are diverse morphologically, histochemically, and physiologically [9]. The most abundant type are the bitufted VIP+ INs that tend to colabel with CR [46, 48], display an irregular-spiking firing pattern near threshold [18, 46, 48, 49, 227], and send a downward projecting axon towards deeper cortical layers. The second most abundant type is the VIP+, CR− bipolar cells, which display a fast adapting firing pattern [44, 46, 48, 227] and send extensively branched projections both locally and towards deep cortical layers. Due to their high input resistance, VIP INs tend to be highly excitable [18, 46, 48]. They have been shown to target pyramidal cell dendrites and somata [17], but some subsets appear to target other interneurons more preferentially [228–230]. The precise function of VIP interneurons in cortical networks remains to be determined. However, their physiological characteristics and diverse synaptic targets render them well suited to rapidly modulate the interactions between pyramidal cells and MGE-derived interneurons. Furthermore, as they receive strong input from pyramidal cells in layers II-III [231], which also receive input from pyramidal cells in other functionally connected cortical areas, VIP interneurons might be important in regulating cross-cortical communication (i.e., sensorimotor modulation where inputs from the sensory cortex modulate the output of cortical motoneurons).

### 1.6. Neurogliaform Cells

Neocortical neurogliaform cells exist in all cortical layers but are the most abundant GABAergic population in superficial layer I [46, 48]. They express reelin (as well as alpha actinin 2 in the rat [103, 232]), but not VIP or SST [9, 46]. They are morphologically distinct as they have multiple radially oriented dendrites extending from a small round soma, as well as a finely branched dense axonal plexus typically extending well beyond the dendritic tree, giving them a spider web appearance [19, 233]. Neurogliaform cells display late-spiking firing patterns with spike accommodation during sustained depolarisations [19, 44, 45, 48, 234]. They have been shown to elicit slow long-lasting inhibitory events (IPSPs) in pyramidal cells and other interneurons by activating both GABA_A_ and GABA_B_ receptors after nonsynaptic volume release of GABA [233–235]. Some of this tonic inhibition is thought to be mediated through the activation of delta subunit-containing GABA_A_ receptors, which are modulated by neurosteroids [235]. This effect might underlie the antiepileptic effect of steroids used to treat pharmacoresistant epilepsies [236, 237]. Furthermore, neurogliaform cells are extensively interconnected by electrical gap-junction synapses but also contact most other interneurons subtypes via similar electrical synapses [238–240]. They are therefore well suited to shape synchronous cortical oscillations. Finally, some neurogliaform cells release nitric oxide, a potent vasodilator, and may therefore play a role in the neurovascular adjustment of blood flow in the face of cerebral hypoperfusion (i.e., strokes, shock, etc.) [241, 242].

## 2. The Development of Cortical Interneurons Depends on Tightly Regulated Genetic Cascades

Cortical interneurons originate in the ventricular zone of the ventral telencephalic ganglionic eminences [41, 43, 243], migrate tangentially up to the cortex [218, 244], and reach their final destination after radial migration across cortical layers. This is quite distinct from cortical pyramidal cells, which originate from the cortical ventricular zone, migrate radially, and reach their final position after a brief bout of tangential migration [42, 245]. The ganglionic eminences are divided into three different subdomains, the medial (MGE), caudal (CGE), and lateral (LGE) ganglionic eminences, which produce distinct subtypes of interneurons in a temporally dynamic fashion [44–46, 246]. Cortical interneurons originate from the MGE and CGE [41, 43, 243], as well as from the preoptic area [47]. Although this has been debated, it is generally believed that the LGE does not give rise to cortical interneurons, instead generating medium spiny neurons of the striatum, nucleus accumbens, and olfactory tubercules, as well as olfactory bulb and amygdalar interneurons [23, 33, 38, 41, 50, 51].

**Figure 2:**
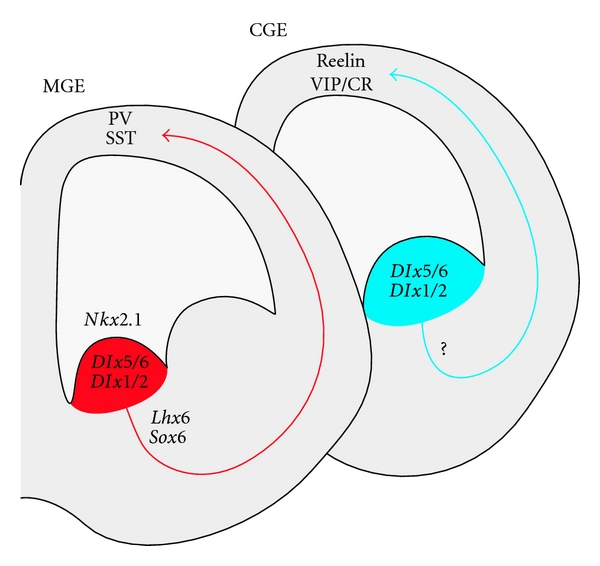
Genetic cascade governing cortical interneuron generation. Corticolimbic interneurons originate in the medial and caudal ganglionic eminences (MGE and CGE). The homeobox transcription factors *Dlx5/6*, *Dlx1/2* and the proneural gene *Mash1* (not shown) are expressed throughout the ganglionic eminences and are required for the generation of all GABAergic interneurons. The MGE generates parvalbumin-positive (PV) basket cells and chandelier cells, as well as somatostatin-positive (SST) cells (including Martinotti cells). These rely on the sequential expression of *Nkx2.1*, *Lhx6*, and *Sox6* for proper specification and maturation (see text). The genetic cascade governing the specification of CGE-derived interneurons has not been fully elucidated yet, but *Nkx6.2* and *Gsh2* are expressed in the CGE and might be important players (see text).

The genetic code that governs the generation and specification of cortical interneurons has been extensively studied over the last decade (Figure 2). The *Dlx* homeobox genes, including *Dlx1/2* and *Dlx5/6,* encode a family of transcription factors crucial for the generation, specification, and migration of all interneurons. The proneural gene *mammalian achaete-scute homolog 1* (*Mash1*), which encodes a basic helix-loop-helix transcription factor, is also crucial for these processes. These genes are broadly expressed across the subpallial subventricular zone (SVZ) of the ganglionic eminences [218, 247–249]. In mice carrying compound *Dlx1* and *Dlx2 *knock-out mutations, GABAergic interneurons fail to migrate out of the ganglionic eminences, resulting in striking reductions in cortical and olfactory bulb interneurons as well as abnormal striatal differentiation [23, 219]. Similar results are seen in mice lacking *Mash1* [250]. Interestingly, *Dlx1/2* gene dosage appears to be important, as interneurons in mice carrying a *Dlx1^−/−^; Dlx2^+/−^* genotype displays normal tangential migration to the cortical plate, but shows altered laminar positioning and simplified morphology (long axons and dendrites with few branches) [220]. Furthermore, *Dlx1^−/−^* mutants display selective defects in the dendritic morphology of SST+/CR+ interneurons, with a progressive loss of these interneurons in the postnatal brain, resulting in spontaneous seizures [221].

The MGE and CGE give rise to distinct cortical interneuronal populations [25, 41]. The MGE generates the parvalbumin-positive (fast-spiking basket cells and chandelier cells) and somatostatin-positive interneurons (including Martinotti cells) [8, 9, 22, 25, 41, 44, 45, 251]. The specification of these interneurons relies on the expression of *NK2 homeobox 1* (*Nkx2-1*) [22, 222]. The loss of *Nkx2-1* as interneuron progenitors are exiting their last mitotic division in the ganglionic ventricular zone leads to respecification of these cells into CGE-type interneurons (of all major subtypes) and the consequent absence of cortical PV and SST interneurons [22]. Interestingly, PV interneurons originate mainly from the ventral MGE whereas SST cells are preferentially produced by the dorsal MGE [251, 252], a phenomenon likely mediated by the combinatorial expression of particular transcription factors within different subdomains of the MGE [252, 253], resulting in part from a gradient of SHH expression [254]. Furthermore, a portion of the dorsal MGE and the MGE-CGE sulcus region is delineated by the expression of the homeodomain transcription factor *Nkx6-2*, which partially overlaps with *Nkx2-1*. This area gives rise to the subgroup of somatostatin cells (about 30%) that coexpress somatostatin and calretinin and display a delayed nonfast spiking firing pattern [251, 255]. 

As they leave the ventricular zone, MGE-derived interneurons begin to express the transcription factor *LIM homeobox protein 6* (*Lhx6*), which is expressed into adulthood [26, 222, 251, 256, 257]. *Lhx6 *is required for proper specification and migration of MGE-derived interneurons, and the loss of *Lhx6 *results in misspecified hippocampal and cortical INs. These cells retain their GABAergic identity, but they fail to express PV or SST and are mislocalised within the neocortex [257]. Indeed, *Lhx6* loss disrupts the correct expression of downstream effectors known to be important for IN migration, including *v-erb-a erythroblastic leukemia viral oncogene homolog 4 *(*ErbB4*), *C-X-C chemokine receptor type 4* (*CXCR4*) and *type 7* (*CXCR7*), and *aristaless-related homeobox* (*ARX*) [26, 258]. 

Downstream of *Lhx6* is *SRY-box 6* (*Sox6*), another transcription factor expressed by MGE-derived interneurons as they initiate their tangential migration. Sox6 is required for the proper laminar distribution and maturation of MGE-derived interneurons [28]. Its loss results in mislocalised MGE-derived INs that accumulate ectopically in layer I and deep layer VI, failing to adequately populate cortical layers II–V [28, 223]. Furthermore, these cells fail to express their mature markers, leading to a striking loss of cortical PV- and SST-expressing cells (PV being more severely affected). Although they remain correctly specified as MGE-INs, as evidenced by their morphology, electrophysiological properties, and expression of GABA, the resulting mutant cells fail to acquire mature intrinsic properties. For instance, PV-cells are unable to sustain the high frequency firing rates expected from these cells by P17-18 [28]. This results in a severe developmental epileptic encephalopathy with early lethality during the 3rd postnatal week [28].

As detailed above, the CGE produces a great variety of cortical interneurons, which populate the more superficial cortical layers. CGE-derived INs include all VIP- and reelin-positive cells, including the calretinin bipolar and neurogliaform cells, as well as multiple smaller subgroups of cortical interneurons, which are distinguishable by their morphological and physiological properties [25, 43, 44, 46]. The master regulatory genes for CGE cell-fate determination have yet to be fully determined. However, some transcription factors are expressed in both the CGE and dorsal MGE, including *Nkx6-2* and *CoupTF1/2*, and might play a role in the specification of CGE interneurons [255].

## 3. GABAergic Interneurons Play Fundamental Roles in Developing Circuits

GABA signalling is crucial during embryogenesis for both neural and nonneural populations of cells [259]. In fact, early GABAergic signalling has been shown to affect neurogenesis, differentiation, migration, and integration of developing neurons into neuronal circuits [260, 261]. Indeed, GABA_A_ receptors are expressed early in newborn pyramidal neurons, which receive GABAergic inputs long before forming excitatory synapses [262, 263]. GABA is excitatory in immature neurons due to the high level of *NKCC1* expression. *NKCC1* increases the intracellular concentration of Cl^−^, shifting the GABA equilibrium potential (E_GABA_) to more depolarised levels, thereby leading to an extrusion of negatively charged chloride anions upon activation of GABA_A_ receptors and a depolarisation of the cell membrane [264]. With time, the progressive expression of another chloride transporter, KCC2, lowers the baseline intracellular concentration of Cl^−^ and underlies the developmental switch of E_GABA_ in favour of an inhibitory effect of GABA in mature neurons [265, 266]. 

This developmental switch is important in controlling the migration, final position, and morphological maturation of interneurons. Tangentially migrating interneurons have been shown to release GABA in a nonvesicular manner [267]. GABA then acts synergistically with AMPA/NMDA receptor-mediated currents to promote tangential migration of interneurons as long as it is depolarising. However, the gradual expression of KCC2 shifts the reversal potential of GABA and the resulting hyperpolarisation acts as a stop signal to arrest the migration of cortical interneurons [268]. Interestingly, interneurons derived from the MGE, which reach their final layer earlier than CGE-derived interneurons, also appear to express KCC2 earlier than CGE cells born simultaneously [246]. KCC2 might therefore regulate some of the differences observed in the laminar distribution of interneurons originating from different sources. 

Furthermore, GABA-mediated depolarisations have recently been shown to promote excitatory synapse formation by facilitating NMDA receptor activation in cortical pyramidal neurons [269]. Blocking these GABA-mediated depolarisations, by *in utero* knock-down of NKCC1 or with the NKCC1 antagonist bumetanide, results in decreased numbers of functional excitatory synapses [269]. These manipulations also lead to altered cell morphologies, including thinner apical dendrites, simplified dendritic trees, and decreased dendritic spine densities [269]. These detrimental effects of bumetanide appear to be long-lasting, as they persist in the adult cortex and are associated with developmental delay and altered prepulse inhibition in adult mice [270]. Premature overexpression of KCC2 leads to similar dendritic anomalies in cortical pyramidal cells as those reported after blocking NKCC1 [271]. 

In summary, GABA plays fundamental roles at different stages of neuronal development, affecting migration, maturation, and synapse formation of both pyramidal cells and interneurons. Furthermore, the precise effect of GABA postsynaptically is dependent on the intracellular concentration of chloride, which is developmentally regulated via the expression of various chloride cotransporters and which varies depending on the age of the cells.

## 4. Interneurons and Early Network Activities

GABergic INs serve diverse functions in developing and in mature networks. As detailed above, they provide local circuit inhibition and participate in the genesis and organisation of specific mature neocortical and limbic oscillations, which in turn modify how local circuits respond to incoming signals. In addition, GABAergic interneurons are critical for the proper maturation and wiring of developing networks [8, 272], as well as for the regulation of critical period experience-dependent cortical plasticity [132, 273–276]. In particular, they have been involved in the generation of some of the early postnatal cortical and limbic oscillatory activities appearing during the first postnatal week in rodents. These synchronised network activities are thought to be important for the proper morphological maturation of excitatory and inhibitory neurons, including for the development of complex dendritic trees and synaptic contacts. 

The first postnatal activities recorded are the synchronous plateau assemblies (SPAs), which are prolonged gap-junction-mediated calcium plateaus appearing between P0-P3 in the rat hippocampus [277] and neocortex [278]. The cellular substrates that drive these SPAs are still unknown, but it is interesting to note that some subsets of cortical interneurons are extensively interconnected through gap junctions [69, 70, 101] and could contribute to the generation of SPAs. In the cortex, SPAs progressively coexist with cortical early network oscillations (cENOs) between P0-P5. cENOs are infrequent (0.01 Hz) synaptically driven calcium events with slow kinetics that depend on glutamatergic AMPA- and NMDA-mediated synaptic activity and that cause sustained depolarisation of large groups of neurons. 

These early network activities are then replaced by the giant depolarising potentials (GDPs) recorded in the hippocampus [279] and neocortex [278] between P6-P8. GDPs are much more frequent (mean 0.1 Hz), consist of fast calcium events, and are entirely dependent on GABAergic synaptic activity (as they are blocked by the GABA_A_ antagonist bicuculline). In the hippocampus, GDPs have been shown to result from the spontaneous activity of a subset of highly connected GABAergic neurons, the hub neurons, that pace whole populations of pyramidal cells in a rhythmic fashion [280]. These hub cells receive more excitatory inputs (EPSPs), display a lower action potential threshold, and have a wider axonal arborisation than neighbouring local GABAergic interneurons [280]. These characteristics render them particularly well suited to generate waves of activity in wide sets of neurons upon stimulation by incoming inputs. GDPs coincide with a phase of active synaptogenesis within the developing neocortical and limbic circuits. It is therefore likely that a selective dysfunction of GABAergic interneurons in these early developmental steps might alter the process of synapse formation, either by decreasing these early network activities or by exerting more direct effects on the postsynaptic membrane. 

In summary, interneurons participate in the genesis of early network activities which provide critical input for the normal maturation and plasticity of corticolimbic networks. In the mature brain, they provide local circuit inhibition and govern the onset and maintenance of some of the corticolimbic oscillations. These combined functions underlie the extensive impact of interneuronopathies on neurodevelopment and cognition.

## 5. Interneuronopathies and Neurodevelopmental Disorders

Interneuron anomalies have been suspected to underlie a variety of neurodevelopmental disorders in humans, including epilepsy, autism, and schizophrenia [14, 15]. This hypothesis stemmed from the observation of decreased GAD67 expression in postmortem brain tissue from affected individuals [110]. Later genetic studies also supported this hypothesis as variants in the GAD67 promoter area were discovered in patients with childhood-onset schizophrenia [113] or bipolar disorder [281]. Interestingly, many other genes linked to neuropsychiatric disease have since been shown to be preferentially expressed in developing cortical interneurons in mice [282]. It is therefore appealing to consider the possibility that genetic anomalies known to affect the development or function of interneurons in mice might be involved in neuropathologies in humans. Although genetic anomalies may manifest differently in mice and humans due to differences in expression patterns or compensation by other genes across species, alterations in highly conserved genetic pathways or disturbances in fundamental physiological processes might translate similarly in humans and mice. Furthermore, a host of environmental factors will likely modify the disease expression in these highly heterogeneous and likely polygenic pathologies. An exhaustive review of the genetic causes of schizophrenia, autism, and epilepsy is beyond the scope of this paper, but we will attempt to summarize some of the compelling evidence pointing to the roles of GABAergic neurons in these disorders.

### 5.1. Interneuron Development in Humans

Human GABAergic interneurons appear to be highly diverse as initially recognized by Ramon y Cajal [283, 284], with a similar array of PV-positive basket cells, PV-positive chandelier cells, SST-positive Martinotti cells, VIP-CR bitufted cells, VIP bipolar cells, and neurogliaform cells as that described in other species [284, 285]. However, the relative proportion of these various populations varies across species [286]. The superficial cortical layers II-III are considerably larger in the human cortex, presumably underlying some of the enhanced intercortical connectivity mediating higher brain functions in primates. Consequently, CR-positive double-bouquet INs appear to be considerably more numerous in the human cortex [284, 285]. Furthermore, although most cortical GABAergic interneurons develop in the ventral ganglionic eminences in humans, a proportion of cortical INs appear to originate from the cortical ventricular zone [284, 287–289]. Nonetheless, similarities do exist with regards to the molecular pathways involved in cortical interneuron genesis in humans and rodents, with preservation of some of the same fundamental genes including *Mash1*, *Dlx1/2*, *Nkx2-1*, and* Lhx6* [288, 290–293].

### 5.2. Interneurons and Schizophrenia

Schizophrenia is a chronic psychiatric condition that combines neurocognitive dysfunctions (i.e., delusions, hallucinations, and disorganisation of thought), negative symptoms (i.e., flat affect, avolition, and alogia), and social or occupational deterioration (i.e., altered social interactions, deterioration in personal hygiene, and inability to self-sustain) [294]. This is accompanied by more specific cognitive impairments such as abnormalities in perception, inferential thinking, volition, linguistic fluency, attention, executive functions (planning), and working memory [295, 296]. 

The involvement of interneurons in the pathophysiology of schizophrenia was suggested when the number of prefrontal cortical GAD67-expressing cells was found to be decreased in autopsy specimens from schizophrenic patients [109, 110]. There is no net loss of cortical PV-positive interneurons or calretinin-positive interneurons in schizophrenic cortices, as the total number of cells stained for either marker is preserved [111, 112]. However, there appears to be a selective downregulation of GAD67 in PV-positive interneurons in schizophrenic brains [112]. Furthermore, the level of parvalbumin expression in these cells is decreased [112]. As both parvalbumin and GAD67 expression are known to be regulated by cortical activity [297, 298], these findings could reflect secondary changes in response to altered levels of cortical activity in schizophrenic patients. Indeed, two schizophrenia susceptibility genes encoding the trophic factor neuregulin 1 (*NRG1*) and its receptor *ErbB4 *(*ERB4*) [117–121] have been shown to facilitate activity-dependent GABA release from PV-positive basket cells in the mouse prefrontal cortex [299]. Selective loss of ErbB4 in PV cells causes a disinhibition of prefrontal pyramidal cells and results in a schizophrenia-like phenotype in mice [126]. In addition, the specific expression of ErbB4 in PV cells is required for neuregulin-1-dependent regulation of hippocampal long-term potentiation [127], which is altered in schizophrenic patients. Interestingly, hypostimulation of PV-positive basket cells via selective ablation of the NR1 subunit of the NMDA receptors in these cells resulted in schizophrenia-like behaviors (working memory deficits, impaired prepulse inhibition, locomotor hyperactivity, and anxiety) and decreased PV and GAD67 expression in PV basket cells in a mouse model of schizophrenia [128]. Therefore, hypofunction of prefrontal PV INs, either through a primary dysfunction of these cells or a decreased excitatory drive to these cells, appears to result in behavioural consequences in mice, which recapitulate aspects of the phenotype observed in schizophrenic patients.

Additionally, other genetic anomalies found in schizophrenic patients that are predicted to affect cortical maturation more broadly appear to impact interneuron maturation and GAD67 expression. For instance, brain-derived neurotrophic factor (BDNF) is normally released in an activity-dependent fashion from pyramidal cells and was shown to regulate the maturation of GABAergic INs [129]. Both BDNF and its receptor TrkB have been found to be downregulated in the prefrontal cortex of schizophrenic patients [122–124]. Knock-out mice for both BDNF and TrkB display behavioral anomalies and a decrease in the synaptic expression of GAD67/GABA [130, 131]. Similarly, the neural cell adhesion molecule NCAM, important for neuronal morphological maturation and synapse formation, requires the addition of a polysialic acid (PSA) moiety to function properly. The activity-mediated expression of PSA has been shown to regulate PV-positive basket cell maturation and determine critical-period plasticity [132]. Interestingly, this PSA-NCAM coupling has been reported to be decreased in hippocampal specimens from schizophrenic patients [125], which would suggest abnormalities in interneuron maturation and cortical plasticity. 

Another interesting hypothesis is that PV-positive chandelier cells might be affected in schizophrenic brains. Indeed, a specific loss of more than 40% of the axonal cartridges (the GAT-1 positive axonal branches from chandelier cells which contact the axon initial segments of pyramidal cells) has been demonstrated in the prefrontal cortex of schizophrenic patients [114, 115]. This is accompanied by enhanced expression of the alpha2 GABA_A_ receptor subunit on the axon initial segment of pyramidal cells, likely as a compensatory mechanism for the decreased input from chandelier cells [300]. However, since chandelier cells are possibly excitatory [94, 95], the net effect of these structural changes on local cortical excitability is uncertain. More recently, the levels of SST, NPY, and CCK were shown to be decreased in a microarray analysis of prefrontal cortical samples from schizophrenic patients [116] (Table 1). Furthermore, there seems to be a specific decrease in SST-positive interneurons, as shown by *in situ* hybridisation staining, in these samples [116]. However, these results await replication. 

Nonetheless, even if the numbers of various interneuron subtypes are preserved and if the morphological structure of these cells is intact in most cases, functional abnormalities in the connectivity of GABAergic circuits likely play a role in the pathogenesis of psychiatric disorders. Modifications of the specific GABA_A_ receptor subunits expressed in the prefrontal cortex of schizophrenic patients have been described [116, 301]. Furthermore, cortical prefrontal gamma oscillations triggered by working memory tasks and selective attention in humans and primates [79–81] are decreased in schizophrenic patients with working memory deficits. These patients display a loss of gamma oscillation power and gamma oscillations are less tightly phase-locked to the task [82, 84, 85]. These changes might reflect functional alterations in the PV-positive basket cells, which contribute to the generation and regulation of the gamma oscillations that synchronise assemblies of pyramidal cells involved in a specific task [69, 72, 76–78, 83, 302]. In summary, multiple studies point to putative anomalies, either structural or functional, in PV-positive INs in the prefrontal cortex of schizophrenic patients.

### 5.3. Interneurons and Autism

Autism is a neurodevelopmental disorder combining impairments in socialization, communication, and restricted interests and/or stereotyped behaviors [294]. Autistic traits can be found in a variety of well-defined neurogenetic syndromes, including tuberous sclerosis [303, 304], fragile X syndrome [133, 134], and Rett syndrome [294]. In addition, nonsyndromic autism (re: without a clear underlying pathology, dysmorphic traits, or structural brain anomalies) has been associated with a variety of *de novo* copy number variants (CNVs) in large genome-wide association studies [141, 305–308], a finding which must be interpreted with caution [309]. However, the discovery of point mutations in genes encoding various synaptic scaffolding proteins in patients with nonsyndromic autism has begun to shed light on the pathophysiology of this disorder (recently reviewed in [309]). In particular, the discovery of mutations in postsynaptic neuroligins (*NRL4X*, *NRL3*) [135, 136], in other postsynaptic scaffolding proteins (*SHANK2*, *SHANK3*) [137–140, 310], in the presynaptic neurexins (*NRXN1*) [141, 142], and in fragile X mental retardation protein (*FMR1* gene) suggest that dysfunction in the maintenance of excitatory synapses, synaptic plasticity, and long-term depression participate in the neurobiology of autism and that this might be rescued by metabotropic glutamatergic antagonists [151–154, 156, 311, 312]. 

In parallel, a dysfunction in GABAergic signalling has been postulated to contribute to the emergence of autistic behaviours. In fact, epilepsy is a frequent comorbidity of autism. Interictal epileptic activity is recorded on scalp EEG in up to 85% of autistic children, although seizures occur in only ~30% of patients [313, 314] (Table 2). This, together with the finding of decreased cortical GAD67/GAD65 expression in autistic patients' brains [143], has suggested that inhibitory dysfunction might play a role in subsets of autistic patients. Furthermore, polymorphisms in the *Dlx1/2* genes have been associated with an increased susceptibility for autism [144] supporting the link between GABAergic anomalies and autism. In addition, nonsyndromic autism has been repeatedly associated with maternal chromosomal duplications in the 15q11-13 region [145, 146], which includes multiple genes encoding various GABA_A_ receptor subunits (*GABRA5*, *GABRG3*, and *GABRB3*). Interestingly, MecP2, a transcription factor that broadly regulates gene expression by binding methylated CPG islands and which is responsible for the majority of cases of Rett syndrome (see next section), also exerts epigenetic control over this chromosomal region [157]. The loss of MecP2 results in dysregulation of multiple genes, including the downregulation of *GABRB3*. Furthermore, the loss of MecP2 is particularly detrimental to interneurons and a conditional MecP2 ablation in GABAergic neurons in mice was recently shown to recapitulate most of the behavioral anomalies associated with Rett syndrome, including autistic-like behavior [158]. 

Finally, another well-characterised mouse model of autism, the *uPAR^−/−^* mouse, displays a spatially selective defect in interneuron migration, such that the frontoparietal cortices of these mice show 50% less calbindin-positive interneurons (with a near absence of PV cells) whereas more caudal cortices are spared [11, 12]. These mice display autistic-like behaviors with increased anxiety and altered socialisation, as well as interictal epileptiform EEG activity and an increased susceptibility to seizures [11, 12]. *uPAR* encodes an urokinase plasminogen activator which is required for the proper processing of the hepatocyte growth factor (HGF). In turn, HGF, through its receptor MET, has been shown to be a critical motogen for interneuron migration and is able to rescue the interneuron migration defect and seizure susceptibility of *uPAR^−/−^* mice [159, 160]. Interestingly, polymorphisms in the MET promoter have recently been described to confer an increased susceptibility to autism and this gene is included in one of the genomic sequences linked to autism susceptibility (7q31) [149, 150]. Autism is a complex disorder and alterations in other GABAergic circuits, including the striatocortical circuits, likely contribute to this behavioural phenotype. Indeed, an interneuron-selective ablation of MET results in decreased cortical PV cells, but massively increased dorsal striatal PV interneurons, leading to a disruption in striatal-mediated procedural and reversal learning [161]. Nonetheless, cortical and hippocampal GABAergic deficits certainly play a role in some of the cognitive-behavioral manifestations of autism, as well as in the associated susceptibility to seizures.

### 5.4. Interneurons and Epilepsy

Perhaps one of the most intuitive consequences of interneuron dysfunction is the development of epilepsy. Multiple mouse models carrying interneuronopathies have been shown to develop seizures [22, 28, 57, 63, 206, 207, 221]. In parallel, various reports point to probable GABAergic interneuron dysfunction in developmental and symptomatic (posttraumatic or poststatus epilepticus) epileptic disorders in humans [315–318]. In most situations, early developmental interneuron anomalies might contribute to seizure disorders both by altering the normal development of cortical circuits, as detailed above, and by failing to provide the acute inhibition required to control excessive excitation in the mature network. Paradoxically, in a state of chronic excitation, INs have been shown to contribute actively to ictogenesis when GABA becomes depolarizing due to the failure of chloride extrusion from damaged neurons [319, 320]. Therefore, both a primary dysfunction of GABAergic inhibitory transmission and a secondary switch to excitatory GABAergic transmission could contribute to the pathogenesis of epilepsy. Understanding the molecular mechanisms governing interneuron development, maturation, and normal function would therefore be very informative in our quest to comprehend human epileptic disorders.

Epilepsy is a heterogeneous disorder, and most cases are symptomatic of focal or widespread CNS lesions (e.g., malformations, tumors, infections, trauma, strokes, hypoxia, etc.). INs dysfunctions might contribute to seizure disorders following such insults, as suggested by the finding of limbic interneuronal loss after brain trauma or prolonged seizures [321–324]. Hippocampal somatostatin-positive interneurons appear to be particularly sensitive to seizure-induced damage as demonstrated in animal models of drug-induced epilepsy [13, 108, 324, 325], as well as in patients with chronic temporal lobe epilepsy [326]. This might point to a more selective vulnerability of this cell type which could be amendable to neuroprotective therapies. A loss of hippocampal PV cells [327] and alterations in the axonal projections of PV-positive chandelier cells have also been reported in patients with chronic epilepsy [316, 325, 328, 329]. Although it is not clear if these changes are the cause or the consequence of repeated seizures [330, 331], they probably contribute to the chronicity of the disease.

### 5.5. Interneurons in Genetic Developmental Epilepsies

Perhaps most interestingly, GABAergic interneuron dysfunction might contribute to a subset of genetic developmental epilepsies. In those cryptogenic epilepsies where no apparent etiology is found on examination or imaging (re: no dysmorphic traits or neurocutaneous stigma and normal brain CT/MRI), but where patients present clear neurological dysfunction as episodic seizures with or without interictal cognitive impairment, an underlying circuit dysfunction is postulated. These patients with severe developmental epilepsies (i.e., Ohtahara syndrome, West syndrome, Lennox-Gastaut syndrome, Dravet syndrome, etc.) are rarely amendable to surgical interventions, and only few reports of neuropathological examination of surgical or postmortem specimens are available. In most cases of West syndrome, the neuropathological evaluation reveals either focal cortical malformations or diffuse brain damage [332–335] but it is found to be “normal” in up to 45% of cases [336]. Nonetheless, functional inhibitory defects with disrupted GABA_A_R function or immature patterns of GABA_A_R subunit expression have been demonstrated in some cases of infantile spasms [318, 337]. Such inhibitory defects might arise as a consequence of genetic mutations that disrupt genes critical for proper interneuron generation or function. For instance, mutations in the alpha1 subunit of the voltage-gated sodium channel Na_V_ 1.1 (*SCN1A*), the *aristaless-related homeobox transcription factor* (*ARX*), the *cyclin-dependent kinase-like 5* (*CDKL5*), various GABA_A_ receptor subunits and in the alpha 1 subunit of the voltage-dependent P/Q-type Ca^2+^ channel (*CACNA1A*) have been described in patients with a variety of epileptic disorders and similar mutations have been shown to impair GABAergic signalling in rodents (Table 3). 

#### 5.5.1. SCN1A

Mutations in *SCN1A*, which encodes the neuronal voltage-gated sodium channel Nav1.1, have been found to underlie a majority (75–85%) of cases of severe myoclonic epilepsy of infancy (Dravet syndrome) [162–166]. Interestingly, *SCN1A* mutations have also been found to cause generalised epilepsy with febrile seizures (GEFS) as well as a variety of disorders with neurocognitive impairment and variable seizure susceptibility [165, 167–171]. This extended phenotypic variability stems both from the nature of the mutations (nonsense mutations cause Dravet syndrome whereas missense mutations tend to cause different phenotypes depending on their location [166, 338–340]) and from the coexistence of genetic modifiers in other genes [172, 173]. Although Nav1.1 channels are found in most neuronal populations in the rodent brain, their loss was found to result in a more selective impairment of interneuronal transmission in mice [199, 200]. Nav1.1 channels tend to cluster predominantly at the level of the axon initial segment of PV-positive interneurons [341], and their loss results in failure of PV cells to maintain high frequency firing rates [341]. By contrast, pyramidal cell transmission is relatively well preserved in Nav1.1 mutants, presumably though compensation by other channels. Therefore, dysfunctions of INs might contribute significantly to the onset of epilepsy in *Scn1a* mutants.

#### 5.5.2. ARX

In a similar fashion, mutations in the *ARX *gene are associated with a variety of neurological syndromes that combine epilepsy and various degrees of cognitive disabilities. The spectrum of phenotypes associated with *ARX* mutations extends from severe X-linked lissencephaly with ambiguous genitalia and severe myoclonic encephalopathies (Ohtahara syndrome, West syndrome), to isolated nonsyndromic mental retardation [175]. The *ARX* gene is necessary for proper neural proliferation, migration, and differentiation [201–203, 342]. In particular, *ARX *was shown to be essential for proper migration and laminar positioning of interneurons [203, 204], partly because it is a direct downstream target of *Dlx1* [205]. Interestingly, *ARX *knock-in mice carrying trinucleotide repeat insertion mutations recapitulating mutations found in IS cases, display decreased numbers of telencephalic NPY+ and calbindin+ interneurons, and present an epileptic phenotype with early epileptic spasms [206]. Furthermore, a conditional deletion of *ARX *in GABAergic interneurons leads to a similar loss of interneuron migration and is sufficient to cause a developmental epileptic phenotype including brief spasm-like seizures [207]. This supports the hypothesis that even if *ARX* mutations might have broader consequences for cortical development, the specific effect on IN migration is fundamental to the development of epilepsy.

#### 5.5.3. CDKL5/MECP2

Other patients with early epileptic encephalopathies have been found to carry mutations in *CDKL5* [176–182], a protein kinase highly expressed in developing and mature neurons [343]. Interestingly, CDKL5 can directly bind and phosphorylate MecP2 and is coexpressed with MecP2 in cortical neurons [208, 209]. In turn, MecP2 is a transcription factor that broadly represses gene expression by binding methylated CPG islands [210, 211] and is therefore involved in the epigenetic control of gene expression. *MECP2* mutations explain a majority of cases of Rett syndrome [147, 148], a neurodevelopmental disorder manifested by progressive microcephaly, developmental regression, stereotypies, and epilepsy. Interestingly, an interneuron selective ablation of *MecP2* recapitulates most of the neurological and behavioral consequences of *MecP2* knock-out mutations in mice [158]. Since MecP2 is a direct downstream target of CDKL5, it is possible that interneuron dysfunction also contributes to the cognitive and epileptic phenotype seen in both *CDKL5* and *MecP2 *mutants.

### 5.6. Voltage-Gated Ca^2+^ Channels

Finally, patients with idiopathic generalized epilepsy syndromes (IGE) have been shown to carry mutations in various GABA_A_ receptor subunits [174, 183–186, 344], as well as mutations or polymorphisms in multiple subunits of voltage-gated calcium channels, including the *CACNA1A*, *CACNB4*, and *CACNA1H* genes [187–191, 345]. These patients present various combinations of myoclonus, generalised tonic-clonic “grand-mal” seizures, and absence seizures “petit-mal.” Mutant mice carrying loss-of-function mutations in *Cacna1a *or* Cacnb4 *display similar generalised spike-wave absence seizures and have been instrumental in advancing our understanding of generalised epilepsies [212, 213, 216, 346]. In these models, an enhanced thalamocortical rebound bursting due to a gain in low-voltage activated Ca^2+^ currents and excessive thalamic GABA_A_ signalling have been shown to result in hypersynchronisation of the thalamocortical circuitry and absence seizures [214, 215, 217, 347]. In addition, we recently demonstrated that selective loss of *Cacna1a* from cortical and limbic MGE-derived interneurons in mice is sufficient to create a severe epileptic encephalopathy with multiple types of generalised seizures [63]. We showed that *Cacna1a* loss resulted in unreliable neurotransmission from PV-positive interneurons. Furthermore, we demonstrated that concurrent loss of *Cacna1a* from both MGE-derived interneurons and cortical pyramidal cells results in a milder epileptic phenotype characterised by absence seizures [63]. These findings suggest that, in some cases, alterations in MGE-derived interneuron function might lead to a variety of generalised seizures and that the severity of the phenotype can be modulated by the involvement of other neuronal populations. Concurrent with these observations, various mouse models with either misspecified or immature MGE-derived interneurons have also been shown to develop severe epilepsies. For instance, *Nkx2-1^−/−^* and *Sox6^−/−^* null mutants die embryonically or perinatally due to a variety of craniofacial and lung anomalies [222, 348]. However, conditional mutants lacking either *Nkx2-1* or *Sox6 *in an MGE-specific manner develop generalised seizures during the 2nd or 3rd postnatal week, leading to early lethality [22, 28]. In a similar fashion, *Dlx1^−/−^* mice also develop spontaneous seizures [221].

One of the limitations in extending some of the experimental findings from genetic models of interneuronopathy to human diseases is that most of the transcription factors important for interneuron development and specification are also involved in specification of other organs (bone, skin, cartilage, lung, and thyroid). Mutations in these genes therefore cause multisystemic disorders in which neurological involvement is often overlooked. For instance, human heterozygote mutations in *Nkx2-1* have been described in a variety of clinical disorders affecting the thyroid, the lungs, and the brain, the so-called “brain-lung-thyroid” syndrome [192]. In some cases, truncating mutations result in severe respiratory failure at birth, due to the lack of surfactant proteins, with mild congenital hypothyroidism and neurocognitive anomalies [193]. In other cases, *Nkx2-1* mutations have been described in patients with benign hereditary chorea, a movement disorder occasionally accompanied by intellectual impairment and seizures [194, 195]. In a similar fashion, heterozygous mutations in *Dlx5/6* genes cause craniofacial and limb anomalies (ectodermal dysplasias) [196, 197]. *Sox6* is known to be important for proper cartilage formation [348–350], and one child with craniosynostosis (premature fusion of the cranial sutures) and facial dysmorphisms has been shown to carry a heterozygous mutation in *SOX6 *[198]. However, even when direct inferences cannot be made between mouse mutants and human patients, the study of these animal models is instrumental in clarifying the role of specific interneuron populations in preventing various types of seizures and is critical to our understanding of epileptogenesis.

## 6. Conclusions and Future Perspectives

In summary, GABAergic INs include diverse neuronal populations which present significant heterogeneity in terms of their biochemical, morphological, and physiological properties. The fate of these INs is governed by tightly regulated genetic cascades. Disruption of these genetic programs, or of genes important for the proper specification, migration, maturation, and/or function of these cells, leads to a variety of cognitive, behavioural, and neurological consequences including autistic behaviors and epilepsy in rodents and humans. For this reason, furthering our understanding of interneuron development across mammalian species might become the cornerstone for the subsequent development of improved diagnostic approaches, and hopefully new therapeutic strategies, for patients with a variety of neurodevelopmental disorders. A fascinating example of this is the development of stem cell transplantation in the treatment of epileptic disorders in rodents [351, 352]. Other such innovative therapeutic approaches will likely emerge as the exquisite complexity of cortical interneurons diversity unravels.

## Figures and Tables

**Table 1:** Findings in schizophrenic patients and correlations in mice models.

Findings		References
Humans		

GAD67	↓ GAD67 in prefrontal cortex	Volk et al. [109]
		Akbarian et al. [110]
	Preserved # number of PV cells, cortex	Woo et al. [111]
		Hashimoto et al. [112]
	↓ GAD67 level in PV cells, cortex	Hashimoto et al. [112]
	Association with polymorphisms in GAD67 promoter	Addington et al. [113]
Chandelier	Decrease in chandelier cells cartridges (GAT1+) in prefrontal cortex	Woo et al. [114]
		Volk et al. [115]
SST	↓ levels of SST in microarray analysis and ↓ number of SST cells, prefrontal cortex	Hashimoto et al. [116]
NPY/CCK	↓ levels of NPY and CCK in microarray analysis	Hashimoto et al. [116]
*NRG1*	Susceptibility locus in *NRG1 *	Stefansson et al. [117, 118]
		Zhang et al. [119]
		Yang et al. [120]
*ERB4*	Susceptibility locus in *ERB4 *	Silberberg et al. [121]
BDNF/Trkb	Downregulation of BDNF in prefrontal cortex	Weickert et al. [122]
		Wong et al. [123]
	Downregulation of BDNF and Trkb in prefrontal cortex	Takahashi et al. [124]
PSA/NCAM	↓ PSA-NCAM complexes in hippocampus	Barbeau et al. [125]
Gamma	Gamma oscillations are triggered by working memory tasks + selective attention	Tallon-Baudry et al. [79]
		Howard et al. [81]
	Decreased power of cortical gamma oscillations and phase locking to memory task	Spencer et al. [82, 84]
		Cho et al. [85]

Mice		

*Erb4*	Selective interneuron loss of *Erb4*: “schizophrenia-like behaviors”	Wen et al. [126]
*Erb4/Nrg1*	Erb4 in PV cells is required for Nrg1-dependant regulation of LTP (hippocampus)	Chen et al. [127]
NR1	Selective loss of the NMDAr NR1 subunit in PV cells: decreased excitatory input to PV cells results in “schizophrenia-like behaviors” and ↓ expression of PV and GAD67	Belforte et al. [128]
*BDNF*	BDNF regulates activity-dependant maturation of PV cells Bdnf^−/−^ and Trkb^−/−^: ↓ synaptic GAD67 and GABA and behavioral anomalies	Huang et al. [129]
		Cotrufo et al. [130]
		Hashimoto et al. [131]
PSA/NCAM	Activity-mediated expression of PSA regulates PV cells maturation and visual plasticity	Di Cristo et al. [132]
Gamma	Gamma oscillations are triggered by stimulating PV cells: enhanced performance	Cardin et al. [77]
		Sohal et al. [83]
	Gamma oscillations depend on PV cells-mediated fast-synaptic inhibition	Bartos et al. [72]

**Table 2:** Findings in autistic children and correlations in mice models.

Findings		References
Humans		

*FMR1*	Patients with fragile X syndrome often display autistic traits	Levitas et al. [133]
		Brown et al. [134]
NRL4X/NRL3	Point mutations in NRL4X and NRL3 associated with X-linked autism	Jamain et al. [135]
	Point mutations in NRL4X in nonsyndromic autism	Laumonnier et al. [136]
SHANK3	Mutations in *SHANK3* in nonsyndromic autism	Durand et al. [137]
		Gauthier et al. [138]
		Moessner et al. [139]
SHANK2	Mutations in SHANK2 in nonsyndromic autism	Berkel et al. [140]
*NRXN1*	Mutations in *NRXN1 *nonsyndromic autism	Szatmari et al. [141]
		Kim et al. [142]
GAD65/67	↓ levels of GAD65/67 in cortex	Fatemi et al. [143]
*Dlx1/2*	Polymorphisms in *Dlx1/2* with increased susceptibility to autism	Liu et al. [144]
15q11-13	Maternal duplications in 15q11-13 in nonsyndromic autism	Baker et al. [145]
	including *GABRA5*, *GABRG3*, *GABRB3* (GABA_A_R subunits)	Hogart et al. [146]
*MECP2*	Mutations in *MECP2* explain the majority of Rett syndrome.	Amir et al. [147]
	Patients display autistic behaviors.	Buyse et al. [148]
*MET*	Polymorphisms in MET promoter associated with autism	Jackson et al. [149]
	Susceptibility locus for autism at 7q31 includes MET gene.	Campbell et al. [150]

Mice		

*Fmr1*	*Fmr1 *k/o: behavioral anomalies improve with glutamatergic antagonists	Dolen et al. [151]
		Bear et al. [152, 153]
Neuroligins/ neurexins	NRL1/2 expression in nonneuronal cells trigger synapse formation in presynaptic cells	Scheiffele et al. [154]
	NL-1 overexpression in hippocampal neurons promotes assembly of excitatory and inhibitory synapses and knock-down results in loss of inhibitory > excitatory synapses	Chih [155]
	Presynaptic *β*-neurexin induces GABA and glutamate synapse differentiation in postcell	Graf et al. [156]
	NRL1,3,4 localise at glutamatergic synapses, NRL2 at both excitatory and inhibitory	Graf et al. [156]
MecP2	Binds methylated CPG islands and exerts epigenetic control of *UBE3A* and *GABR3 *	Samaco et al. [157]
	Interneuron selective loss of MecP2 recapitulates the Rett-like behavioral aN in mice	Chao et al. [158]
uPAR, HGF, MET	*uPAR^−/−^* displays 50% loss of IN in cortex and seizure susceptibility	Powell et al. [11]
	uPAR is required for the processing of HGF (an interneuron motogen),	Powell et al. [159]
	HGF, through its receptor MET, can rescue the phenotype of uPAR^−/−^ mice	Bae et al. [160]
	Interneuron selective MET ablation: ↓ PV cortex, ↑ striatal PV cells, disrupts reversal learning	Martins et al. [161]

**Table 3:** Selected examples of genes causing epilepsy in humans and interneuron dysfunctions in mice.

Findings		References
Humans		

*SCN1A*	*SCN1A* mutations explain the majority of Dravet syndrome	Claes et al. [162]; Ohmori et al. [163]
		Sugawara et al. [164]; Orrico et al. [165]
		Escayg and Goldin et al. [166]
	*SCN1A* mutations display phenotypic heterogeneity: GEFS, febrile seizures, cognitive impairment	Escayg et al. [167, 168]
		Fujiwara et al. [169]; Osaka et al. [170]
		Zucca et al. [171]; Orrico et al. [165]
	Variants in other channels modify the phenotype of *SCN1A: SCN8A *	Martin et al. [172]
	* CACNB4*	Ohmori et al. [173]
*SCN1B*	*SCN1B* mutations cause GEFS	Wallace et al. [174]
*ARX*	ARX mutations cause various phenotypes including infantile spasms	Shoubridge et al. [175]
*CDKL5*	*CDKL5* mutations cause early epileptic encephalopathies	Kalscheuer et al. [176]; Weaving et al. 2004 [177]
		Scala et al. [178]; Archer et al. [179]
		Cordova-Fletes et al. [180]; Mei et al. [181]
		Melani et al. [182]
*MECP2*	*MECP2* mutations explain most cases of Rett syndrome. These patients often display seizures.	Amir et al. [147]; Buyse et al. [148]
*GABRG2 *	Mutations in the gamma2 subunit of the GABA_A_R cause childhood absence epilepsy ± febrile seizure	Wallace et al. [174]; Kananura et al. [183]
	Truncation of *GABRG2* causes generalised epilepsy with febrile seizure (GEFS)	Harkin et al. [184]
*GABRA1*	Mutations in the alpha1 subunit of the GABA_A_R cause juvenile myoclonic epilepsy	Cossette et al. [185]
	Mutations in the alpha1 subunit of the GABA_A_R can also cause childhood absence epilepsy	Maljevic et al. [186]
*CACNA1A*	Polymorphisms associated with generalised epilepsy syndromes	Chioza et al. [187]
	Mutations in *CACNA1A* can cause ataxia with generalized seizures	Jouvenceau et al. [188]; Imbrici et al. [189]
*CACNB4*	Mutations in *CACNB4* cause episodic ataxia with generalized seizures	Escayg et al. [190]
*CACNA1H*	Mutations in T-type calcium channel Cav3.2 cause childhood absence epilepsy	Khosravani et al. [191]
*Nkx2.1*	*Nkx2.1* haploinsufficiency leads to the “brain-lung-thyroid syndrome”	Carre et al. [192]
	variable phenotype: severe respiratory distress at birth, mild-moderate hypothyroidism, chorea	Guillot et al. [193]
	Some patients present benign hereditary chorea, occasionally with cognitive impairment and seizures	Kleiner-Fisman et al. [194, 195]
*Dlx5/6*	*Dlx5/6* mutations result in craniofacial and limb anomalies: ectodermal dysplasia	Morasso et al. [196]; Lo Lacono et al. [197]
*Sox 6*	1 patient described with heterozygote *Sox6* mutation: craniosynostosis and facial dysmorphisms.	Tagariello et al. [198]

Mice		

*Scn1a*	*Scn1a* (Nav1.1) expressed in most neuronal populations	Yu et al. [199]
	*Scn1a^+/−^*and *Scn1a^−/−^*mice develop spontaneous seizures and die prematurely	Yu et al. [199]
	↓ sodium currents are specific to GABAergic interneurons in *Scn1a^+/−^* and *Scn1a^−/−^*	Yu et al. [199]
	Selective loss of* Scn1a* in interneurons recapitulates seizure disorder	Martin et al. [200]
*Arx*	Role in neuronal proliferation and migration	Fricourt et al. [201, 202]
	Specific requirement of Arx for interneuron migration	Friocourt and Parnavelas [203]; Poirier et al. [204]
	*Arx* is a downstream target of *Dlx1 *	Colasante et al. [205]
	Arx(GCG)10+7 mice display seizures including spasms and ↓ no. of CB and NPY interneurons	Price et al. [206]
	Selective loss of Arx in interneurons recapitulates the seizure disorder	Marsh et al. [207]
Cdkl5	Cdkl5 is coexpressed with Mecp2 in cortical neurons and can phosphorylate Mecp2	Mari et al. [208], Bertani et al. [209]
MecP2	Mecp2 broadly represses gene expression by binding methylated CPG islands	Nan et al. [210, 211]
*Cacna1a*	*Cacna1a^tg/tg^* tottering mutant displays ataxia and absence seizures	Noebels et al. [212]; Fletcher et al. [213]
	Gain of thalamic T-type currents cause enhanced rebound bursting of TC cells in *Cacna1a^tg/tg^, Cacna1a^ln/ln^*	Zhang et al. [214]; Tsakiridou et al. [215]
	Interneuron selective ablation of* Cacna1a* leads to multiple types of generalised seizures incl. absences	Rossignol et al. [63] (abstract)
*Cacnb4*	* Cacnb4^lh/lh^* loss-of-function mutants display spontaneous absence seizures and ataxia	Burgess et al. [216]
	Thalamic tonic GABA_A_ currents enhance rebound bursting of TC cells in *Cacnb4^lh/lh^*	Cope et al. [217]
*Dlx1/2 *	*Dlx1^−/−^Dlx2^−/−^* mice die perinatally and display a failure of IN migration to cortex and olfactory bulb	Anderson et al. [23, 218]; Bulfone et al. [219]
	*Dlx1^−/−^Dlx2^+/−^* abnormal laminar distribution of IN and simplified morphology	Cobos et al. [220]
	*Dlx1^−/−^* morphological defect and postnatal loss of SST+/CR+ interneurons: spontaneous seizures	Cobos et al. [221]
*Nkx2.1*	*Nkx2.1^−/−^* die perinatally. Nkx2.1 is required for MGE interneuron generation.	Sussel et al. [222]
	Interneuron specific removal of *Nkx2.1* results in misspecification of MGE cells into CGE cells, and seizures	Butt et al. [22]
*Sox6*	*Sox6^−/−^* dies perinatally of craniofacial anomalies	
	Conditional loss of *Sox6* in interneurons results in misplaced/ectopic and immature basket cells (loss PV)	Batista-Brito et al. [28]; Azim et al. [223]
	Conditional loss of Sox6 in interneurons results in a severe epileptic encephalopathy	Batista-Brito et al. [28]
